# Photobiomodulation-Driven Tenogenic Differentiation of MSCs in Hydrogel Culture

**DOI:** 10.3390/ijms262411965

**Published:** 2025-12-12

**Authors:** Brendon Roets, Heidi Abrahamse, Anine Crous

**Affiliations:** Laser Research Centre, Faculty of Health Science, University of Johannesburg, Doornfontein, P.O. Box 17011, Johannesburg 2028, South Africa; broets@uj.ac.za (B.R.); habrahamse@uj.ac.za (H.A.)

**Keywords:** photobiomodulation, tenogenic differentiation, hydrogel, 3D cell culture, tenocytes and iADMSCs

## Abstract

Tendon healing is limited by hypocellularity and low metabolic activity, resulting in poor regeneration. Mesenchymal stem cells (MSCs) offer potential for tendon repair, but reliable tenogenic differentiation protocols remain undefined. Photobiomodulation (PBM) has been proposed as an adjunct to assist differentiation, yet standardized parameters are lacking, particularly in 3D systems. This study evaluated the effects of PBM at 525 nm, 825 nm, and combined wavelengths, delivered at 5 J/cm^2^ and 10 J/cm^2^, on immortalized adipose-derived MSCs (iADMSCs) encapsulated in TrueGel3D hydrogels, with the goal of optimizing parameters to support tenogenic differentiation. Immortalized ADMSCs were characterized by immunofluorescence (CD44, CD90, and CD166) and encapsulated in hydrogels. Following a single PBM exposure, differentiation was induced with transforming growth factor-β1 and ascorbic acid for 3 days, followed by the addition of connective tissue growth factor for an additional 7 days. Morphology, membrane permeability, proliferation, and gene expression were assessed at days 1, 4, and 10. The cells adopted a spindle-shaped fibroblastic morphology, forming dense cellular networks throughout the hydrogel, although without alignment due to random RGD distribution. LDH release remained low across groups, confirming biocompatibility. Proliferation rates were not significantly different on day 1. By day 4, green and consecutive PBM at 10 J/cm^2^ and day 10 green PBM at 5 J/cm^2^ showed increased proliferation rates, respectively. PCR analysis showed co-expression of Scleraxis and Tenomodulin in all groups by day 10, confirming tenogenic differentiation. NIR and consecutive (10 J/cm^2^) PBM maintained Scleraxis expression over time, with NIR PBM enhancing Collagen I, III, Biglycan and Tenascin-C on day 1 and 4. However, consecutive PBM (10 J/cm^2^) maintained higher expression patterns more consistently compared to NIR on day 10. Thus, consecutive (525/825 nm) wavelengths at 10 J/cm^2^ proved effective in enhancing tenogenic marker expression for a single-dose PBM protocol.

## 1. Introduction

Tendon injuries represent a major clinical challenge due to the tissue’s limited intrinsic healing capacity, which is largely attributed to its hypocellularity and low metabolic activity [1,2]. Instead of regenerating native structure, tendon repair is often characterized by scar tissue formation and inferior mechanical function. Current surgical and conservative interventions frequently result in incomplete recovery, underscoring the need for regenerative medicine strategies to restore both structure and function [3]. Among these, the directed differentiation of mesenchymal stem cells (MSCs) into tenocytes has emerged as a promising approach, offering a replenishable source of cells capable of synthesizing tendon-specific extracellular matrix (ECM). However, tendon tissue engineering remains limited by the absence of standardized tenogenic differentiation protocols. Immortalized ADMSC lines such as ASC52Telo are increasingly used in stem cell research because they can be expanded over many passages, pose fewer ethical challenges, and still retain the capacity to differentiate [4]. These lines generally preserve key features of primary ADMSCs, including typical surface marker profiles and gene-expression patterns, making them a reliable and consistent model for early-stage in vitro studies of stem cell behavior and lineage specification [5].

Traditional two-dimensional (2D) culture systems, while widely used, do not adequately replicate the complexity of the tendon niche. This design philosophy is consistent with recent work showing that biocomposite scaffolds facilitate favorable stem cell behavior in three-dimensional systems as compared to two-dimensional cultures [6]. Three-dimensional (3D) culture systems provide a more physiologically relevant microenvironment that enhances cell–cell and cell–matrix interactions, thereby supporting lineage-specific differentiation [7,8,9]. Hydrogels, with their high-water content, tunable mechanical properties, and ability to incorporate adhesion motifs and growth factors, are particularly attractive for tendon tissue engineering [10,11]. TrueGel3D hydrogels, in particular, provide a defined synthetic environment, allow for stiffness and RGD motif customization, and controlled degradability, supporting stem cell viability, proliferation, differentiation, and extracellular matrix (ECM) deposition [12,13,14]. An additional advantage of hydrogel systems is their optical clarity, which allows for efficient penetration of photobiomodulation (PBM) light into encapsulated constructs [15].

PBM has emerged as a non-invasive adjunct that can enhance cellular functions relevant to tissue repair, including proliferation, metabolic activity, and viability [16]. While most PBM studies have focused on osteogenesis and chondrogenesis, there is growing interest in its application to tenogenesis. PBM acts through light absorption by intracellular chromophores, resulting in enhanced mitochondrial activity, ATP production, and modulation of signaling pathways that regulate gene expression [17]. However, no standardized PBM protocol currently exists for tenogenic differentiation, and optimal parameters such as wavelength and fluence remain undefined [18]. Therefore, a single exposure was selected for this study to isolate wavelength- and fluence-specific transcriptional responses without cumulative multi-dose effects.

In this study, we investigated the ability of PBM to enhance tenogenic differentiation of adipose-derived MSCs (AD-MSCs) encapsulated in TrueGel3D hydrogels. To achieve this, we assessed stem cell identity, morphology, membrane permeability, proliferation, and gene expression using MSC characterization, immunofluorescence, lactate dehydrogenase (LDH) quantification, ATP quantification, and quantitative PCR (qPCR), respectively. PBM was applied at 525 nm (green), 825 nm (near-infrared), and consecutive 525/825 nm wavelengths at fluences of 5 J/cm^2^ and 10 J/cm^2^ over a 10-day period. While PBM has been explored in other lineages in 3D, no studies to date have examined PBM-mediated tenogenic differentiation in a 3D hydrogel. The purpose of this study is to optimize PBM parameters for tenogenic differentiation in a 3D hydrogel culture model, thereby establishing a basis for future standardization between experimental protocols in the field of tendon tissue engineering.

## 2. Results

### 2.1. MSC Characterization

**Figure 1:**
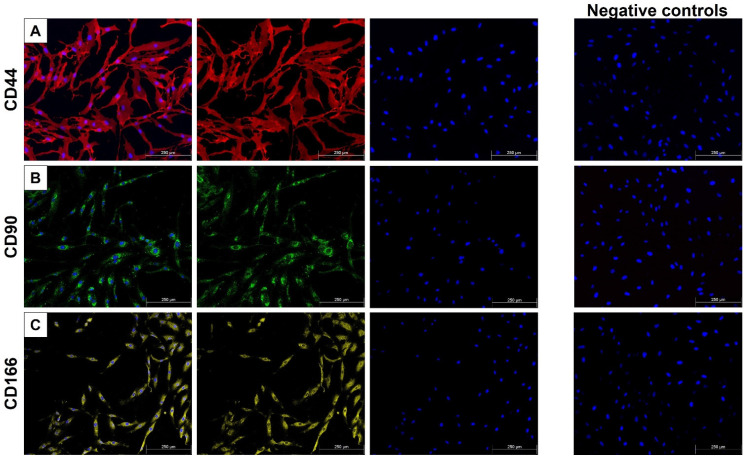
MSC marker expression. (**A**) Positive CD44 expression (red). (**B**) Positive CD90 expression (green). (**C**) Positive CD166 expression (yellow), with their corresponding negative controls. DAPI was used to identify the nuclei of the cells (Blue). Micrographs were produced using the Leica Mica Microhub; magnification of ×10; scale bar is 250 µm.

Immunofluorescent staining demonstrated positive expression of common MSC markers CD44, CD90, and CD166 as seen in Figure 1. The respective negative controls showed no staining, confirming the specificity of the antibodies used.

### 2.2. Morphology

**Figure 2:**
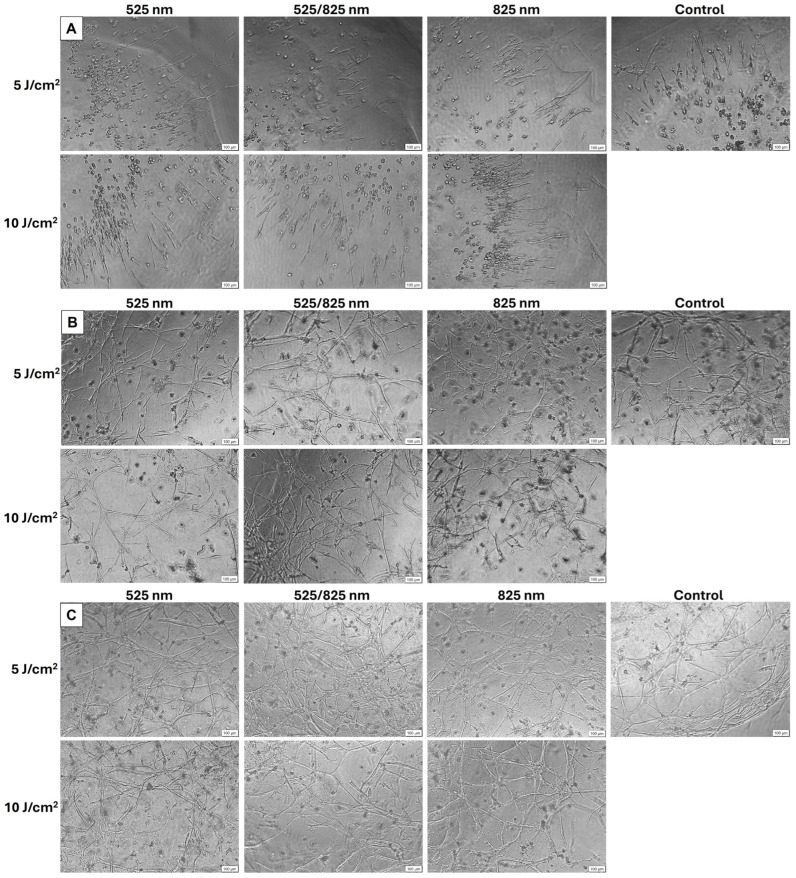
Tenogenic morphology during 3D hydrogel culture. Inverted light microscopy was used to capture the morphology of hydrogel encapsulated iADMSCs during tenogenic differentiation. (**A**) Day 1 after PBM exposure majority of the cells were observed to be round to oval and scattered throughout the hydrogel, with some cells showing signs of spreading. (**B**) Day 4 after PBM exposure majority of the cells assumed a fibroblastic morphology, with some cells remaining round. (**C**) Day 10 after PBM exposure, the cells developed a dense network of interconnected, elongated spindle-shaped cells, with a significant increase in cell number. Micrographs were produced using the Olympus CKX41 light microscope; magnification of ×10; scale bar is 100 µm.

Inverted light microscopy was used for the purpose of monitoring cellular health, distribution, and general morphological progression within the 3D hydrogel, providing visual confirmation that the cells survived encapsulation, adhered within the matrix, and remained viable throughout the culture period. Initially immortalized ADMSCs displayed a predominantly round-to-oval morphology distributed throughout the hydrogel. Day 1 after irradiation, most cells retained this rounded morphology, although some cells began to show signs of spreading and elongation, particularly near the gel periphery (Figure 2A). By day 4, the majority of the cells adopted a spindle-shaped morphology characteristic of fibroblastic cells (Figure 2B). By day 10, the cells formed a dense interconnected cellular network throughout the hydrogel with a significant increase in cell density (Figure 2C). Some cells did not survive the encapsulation process and can be seen as single round cells or cell fragments.

### 2.3. Cellular Health

#### 2.3.1. Membrane Permeability

**Figure 3:**
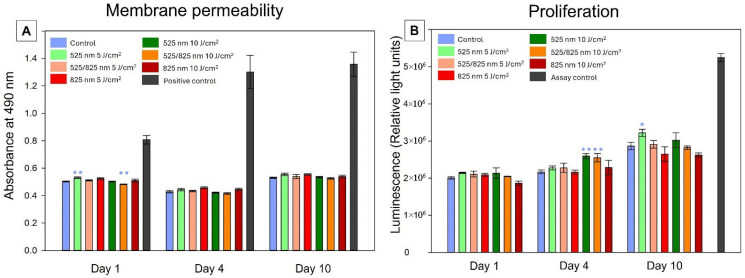
Cellular health in 3D culture during tenogenic differentiation. (**A**) LDH levels were quantified as a measure of membrane permeability. On day 1, green PBM (5 J/cm^2^) had significantly higher and consecutive (10 J/cm^2^) PBM had significantly lower LDH release compared to the day 1 control. By day 4, levels normalized and declined, signaling cellular adaptation to encapsulation. On day 10, LDH levels rose compared to day 1 without significant differences between groups. Positive controls representing maximal cell death were significantly higher than all groups at each respective time point, highlighting the absence of cytotoxicity. (**B**) ATP levels were quantified as a measure of proliferation rates. On day 1, no significant differences were detected across all groups. On day 4, the green and consecutive 10 J/cm^2^ showed significant proliferation rates, compared to the day 4 control. The effects faded out by day 10, with peak rates observed in the green 5 J/cm^2^ group. Data represented as mean ± standard error of quadruplicate experiments (* *p* < 0.05 and ** *p* < 0.01).

LDH release was measured to assess cytotoxicity across treatment groups (Figure 3A). On day 1, the 525 nm, 5 J/cm^2^ group showed significantly elevated LDH release compared to the day 1 control, while the consecutive 525/825 nm at 10 J/cm^2^ group exhibited significantly reduced LDH release. No significant differences were observed in the other groups. By day 4, LDH release decreased across all groups relative to day 1, and no significant differences were detected between treatment conditions. At day 10, LDH levels increased again in all groups compared to day 1; however, no significant differences were found between treatment groups. Importantly, LDH release in all PBM-treated groups remained significantly lower than the positive control at every time point, confirming that neither hydrogel encapsulation nor PBM exposure induced cytotoxicity.

#### 2.3.2. Proliferation

Adenosine triphosphate quantification was used as a measure of proliferation (Figure 3B). On day 1, no significant differences were observed between groups, although slower proliferation was observed in the NIR (10 J/cm^2^) group. By day 4, both green and consecutive PBM at 10 J/cm^2^ showed significantly higher ATP production, compared to the day 4 control. At day 10, green PBM (5 J/cm^2^) showed the highest ATP production. All other groups showed no significance compared to the control.

### 2.4. Gene Expression

Quantitative PCR analysis was used to evaluate the effect of PBM at various wavelengths (525 nm, 825 nm, and 525/825 nm consecutive) and two energy densities (5 J/cm^2^ and 10 J/cm^2^), on the tenogenic differentiation of iADMSCs, cultured in 3D. Expression levels of key tenogenic markers were assessed on day 1, day 4, and day 10, respectively.

**Figure 4:**
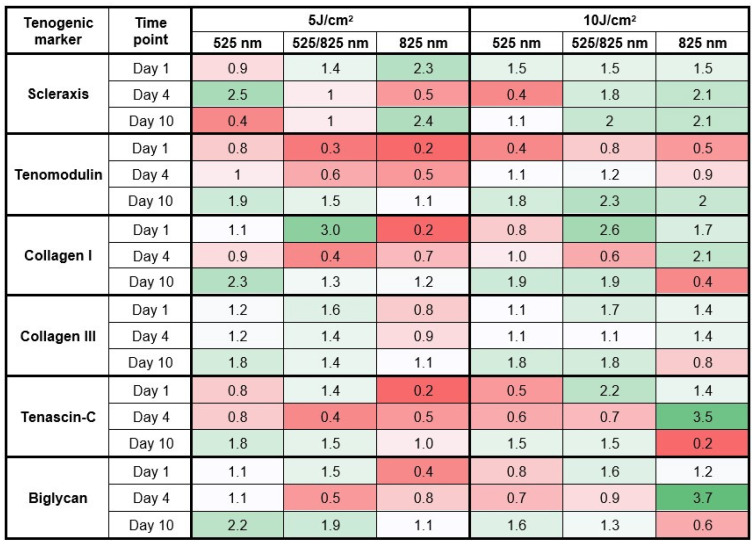
Gene expression patterns of PBM-assisted tenogenic differentiation of iADMSCs in 3D culture. Gene expression fold-changes in key tenogenic markers (Scleraxis, Tenomodulin, Collagen I, Collagen III, Tenascin-C, and Biglycan) (n = 8) were assessed at day 1, day 4, and day 10 following PBM at 525 nm, 825 nm, or consecutive 525/825 nm wavelengths delivered at 5 J/cm^2^ or 10 J/cm^2^. **Day 1:** 825 nm (5 J/cm^2^) strongly induced Scleraxis expression, while tenomodulin remained suppressed in all groups, and consecutive PBM promoted early Collagen I and III expression. **Day 4:** 525 nm (5 J/cm^2^) maximally upregulated Scleraxis, while 825 nm (10 J/cm^2^) strongly enhanced ECM remodeling proteins Tenascin-C and Biglycan. **Day 10:** consecutive (10 J/cm^2^) and 525 nm (5 J/cm^2^) PBM significantly increased Scx and Tnmd expression and TNC and BGN expression. The heatmap uses a red–white–green color scale where red represents suppressed expression, white indicates baseline expression, and green denotes upregulation, with color intensity reflecting the magnitude of change.

At day 1, Scx was strongly upregulated under 825 nm at 5 J/cm^2^ (2.3-fold), while all 10 J/cm^2^ groups gave a moderate induction (1.5-fold) (Figure 4). By day 4, the highest expression was observed with 525 nm at 5 J/cm^2^ (2.5-fold), whereas 825 nm (5 J/cm^2^) decreased expression (0.5-fold), and both 825 nm and consecutive at 10 J/cm2 further increased expression. At day 10, Scx expression stabilized under both 5 and 10 J/cm^2^ 825 nm (2.1–2.4-fold) and consecutive 10 J/cm^2^ (2.0-fold) PBM, while green light alone declined (0.4-fold). Tenomodulin expression was suppressed across all groups at day 1 (≤0.8-fold). At day 4, slight increases were seen with 525 nm and consecutive 10 J/cm^2^ PBM (1.1–1.2-fold). By day 10, strong induction was detected under consecutive (10 J/cm^2^, 2.3-fold), 825 nm (10 J/cm^2^, 2.0-fold), and 525 nm (5 J/cm^2^, 1.9-fold).

Early Col I expression (day 1) was markedly increased using consecutive PBM (5 J/cm^2^, 3.0-fold and 10 J/cm^2^, 2.6-fold). At day 4, levels decreased in most groups, except under 825 nm 10 J/cm^2^ (2.1-fold). At day 10, Collagen I remained elevated under 525 nm (5 J/cm^2^, 2.3-fold and 10 J/cm^2^, 1.9-fold) and 10 J/cm2 consecutive PBM (1.9-fold). Moderate increases in Col III were observed consistently across all groups, ranging 1.2–1.8-fold across time points, with the strongest upregulation at day 10.

At day 1, moderate TNC induction occurred under consecutive PBM at 10 J/cm^2^ (2.2-fold). The most pronounced effect was at day 4 under 825 nm at 10 J/cm^2^ (3.5-fold). By day 10, expression stabilized (1.5–1.8-fold) in most groups but declined in both 825 nm groups (0.2-fold). Similarly, moderate BGN increases were observed at day 1 using consecutive PBM (5 J/cm2,1.5 and 10 J/cm2, 1.6-fold). At day 4, expression was strongly induced by 825 nm at 10 J/cm^2^ (3.7-fold). By day 10, sustained upregulation was observed under 525 nm at 5 J/cm^2^ (2.2-fold) and consecutive PBM at 5 J/cm^2^ (1.9-fold).

## 3. Discussion

In spite of growing interest in the use of regenerative medicine for musculoskeletal repair, there is a lack of standardized tenogenic differentiation protocols and a clear shortage of literature specifically addressing PBM-assisted tenogenic differentiation [19,20]. At present, there is no clear consensus on the selection of PBM parameters in this context, and most studies often focus on PBM use for in vivo tissue repair rather than in vitro tissue engineering, and are limited to conventional 2D culture studies. To the best of our knowledge, no published study has evaluated PBM for tenogenic differentiation within a 3D hydrogel culture model. Furthermore, no study has directly compared green (525 nm), NIR (825 nm), and 525/825 nm consecutive PBM for their effects on tenogenic differentiation. These wavelengths were selected based on prior evidence that NIR PBM enhances proliferation and cell viability, while green light has been linked to promoting differentiation pathways [21,22,23]. The fluences were selected to reflect the established therapeutic window of PBM (1–5 J/cm^2^), while a higher dose (10 J/cm^2^) was included to account for the increased optical scattering and attenuation expected within the 3D hydrogel system, where lower fluences might have been insufficient to elicit biological effects [24,25]. However, the TrueGel3D hydrogel system is optically clear to minimize these effects.

MSCs are characterized by their multilineage differentiation capability and expression of surface markers, most commonly CD44, CD90, and CD166 [26]. Our research unit has previously demonstrated the osteogenic and neuronal differentiation capacity of this cell line, confirming its multipotency [27,28]. Prior to differentiation and irradiation, immunofluorescent staining confirmed the expression of MSC markers CD44, CD90, and CD166, supporting the MSC identity of the starting cell line. After iADMSC encapsulation, morphology was evaluated, since cell shape and organization are closely linked to differentiation and cell health.

The observed morphological changes reflect typical MCS behavior in 3D matrices, where cells initially adopt a rounded morphology due to hydrogel encapsulation constraints [29,30]. By day 4, the cells assumed a spindle-shaped morphology, showing signs of spreading and elongation, as they interact with adhesive ligands and remodel their microenvironment. These features are commonly associated with a fibroblast-like or tenocyte-like phenotype. Furthermore, some cells did not survive encapsulation and can be seen as single rounded cells or cell fragments [31]. By day 10, the cells formed dense interconnected cellular networks randomly orientated throughout the hydrogel [32]. Three-dimensional culture allows these networks to form and improve cellular communication and signaling. The increase in cell density observed across the experimental period further indicates that the hydrogel environment supported survival and proliferation of encapsulated cells [33]. The lack of cellular alignment observed in this study is noteworthy, as aligned morphologies are frequently reported in tendon tissue and are critical for function. This absence can be attributed to the random distribution of RGD peptides within the hydrogel matrix, which provides non-directional adhesion sites [34]. The absence of directional matrix cues represents a significant confounder when interpreting tenogenic differentiation, as tendon cells require aligned ECM structures. This constraint is inherent to isotropic hydrogels and may limit PBM responsiveness due to reduced mechano-signaling. Previous studies have demonstrated that alignment cues such as fiber orientation are necessary to achieve anisotropic organization and parallel alignment of tendon-like cells [35]. Incorporating such cues into future hydrogel designs may better replicate the native tendon microenvironment.

LDH release serves as an indicator of cell membrane damage, with elevated levels reflecting cytotoxicity. The significant increase observed on day 1 in the 525 nm, 5 J/cm^2^ group suggests that low-fluence green PBM may initially induce mild stress or transient membrane disruption. This temporary increase may be attributed to acute photophysical PBM effects, since green PBM is associated with moderate ROS formation [36]. However, this effect was not sustained, as LDH levels normalized by day 4. Conversely, the significant reduction in LDH observed under consecutive 525/825 nm PBM at 10 J/cm^2^ suggests that combined wavelengths at higher fluence may provide a cytoprotective effect [37]. By day 4, all groups exhibited reduced LDH release relative to day 1, reflecting cellular adaptation to the 3D environment, which is consistent with morphological evidence of spreading and proliferation. Similarly, a previous osteogenic differentiation study using PBM and hydrogel encapsulation confirmed the absence of sustained cytotoxic effects [38]. At day 10, LDH levels increased again across groups, which may reflect cumulative metabolic stress, nutrient diffusion limitations, or increased turnover in dense hydrogel cultures [39]. Crucially, there were no significant differences between PBM groups at this stage, indicating that the observed late-stage increase was a general culture effect rather than PBM-specific. Most importantly, LDH release in all PBM-treated groups remained significantly lower than the positive control at all time points, demonstrating that neither PBM nor hydrogel encapsulation induced cytotoxicity [40].

Proliferation of differentiating iADMSCs within the 3D hydrogel environment was generally slower compared to expected growth rates in conventional 2D culture. This is consistent with prior observations that encapsulated cells proliferate more slowly due to restricted spreading and increased reliance on cell–matrix interactions [41]. In addition, differentiation processes inherently reduce proliferation rates, as cellular ATP is increasingly redirected away from mitosis toward lineage-specific specialization and ECM synthesis [42,43]. On day 1, no significant differences were detected between treatment groups. This may reflect an early redirection of ATP utilization away from proliferation and toward differentiation. By day 4, both green and consecutive PBM regimens at 10 J/cm^2^ showed significantly higher proliferation compared to other groups. This result is in agreement with earlier studies reporting that green light can enhance proliferation, typically with peaks observed around day 3–4 of culture [44,45,46]. However, the proliferative effects of single PBM irradiation are often transient and fade during long-term cultures. At day 10, green PBM (5 J/cm^2^) produced the highest proliferation, suggesting that low-fluence green irradiation may be effective in expanding tenogenic cells in 3D hydrogel culture. From a translational standpoint, this is important because tendon tissue is inherently hypocellular and exhibits low proliferative capacity, which contributes to poor healing outcomes [2]. Therefore, PBM can help overcome this limitation by providing a larger pool of metabolically active cells capable of contributing to tendon repair and regeneration. It is important to note that the ATP levels were not normalized to cell count, limiting the ability to distinguish metabolic shifts from changes in cell density. However, taken together, these findings and membrane permeability findings suggest that PBM and hydrogel encapsulation provide a sufficient environment for cells to proliferate and differentiate.

The quantitative PCR results demonstrated that PBM modulates tenogenic differentiation of iADMSCs in a wavelength-, fluence-, and time-dependent manner, based on the observed temporal patterns. Supporting the potential of PBM as a non-invasive strategy to assist tenogenic lineage commitment and functional tendon matrix development. However, further validation on the protein level is required. Early (day 1) responses focused on the induction of Scx, the master regulator of tendon lineage commitment. NIR PBM (5 J/cm^2^) produced the strongest early Scx induction, while all 10 J/cm^2^ groups provided moderate activation. By day 4, Scx expression peaked under green PBM (5 J/cm^2^), suggesting a delayed transient activation. Previously, it has been shown that green PBM can stimulate differentiation pathways in MSCs [21]. By day 10, Scx expression remained stable under NIR and consecutive (10 J/cm^2^) PBM, but declined under green PBM alone, suggesting that NIR wavelengths may potentially sustain transcriptional lineage priming over longer culture periods compared to green PBM [47]. These observations agree with Li et al., who showed that 532 nm PBM upregulated Scx and Tnmd in tendon-derived stem cells via Nr4a1 signaling, and with broader evidence that NIR PBM enhances stem cell differentiation through mitochondrial and ROS-mediated regulation [22,36,48].

Tenomodulin, a terminal marker of tendon maturation, was suppressed during early culture but became strongly expressed by day 10 across groups, particularly under consecutive PBM (10 J/cm^2^) and NIR PBM (10 J/cm^2^) with sustained elevated Scx expression, suggestive of a more effective differentiation. The delayed induction of Tnmd is consistent with its role as a late-stage marker of tenocyte maturation and tendon functionality [49]. Importantly, the co-expression of Scx and Tnmd at day 10 confirms tenogenic characterization of the differentiated cells [50].

Analysis of ECM proteins showed distinct stage-specific regulation. Collagen I, the predominant tendon collagen, was strongly upregulated under consecutive PBM at day 1. Similarly, Lim et al. showed that combining PBM wavelengths improved collagen I/III organization during in vivo studies [51]. However, the effect diminished by day 4, only to reappear by day 10 under green and consecutive (10 J/cm^2^) regimens. Reflecting the ability of both green and NIR PBM to enhance collagen production [52]. However, NIR 10 J/cm^2^ PBM sustained collagen expression across days 1 and 4, indicating that NIR PBM at higher fluence is particularly effective at driving initial matrix production. These results are supported by animal studies reporting that NIR PBM (830 nm) enhanced both type I and III collagen synthesis in tendon repair models [53]. Collagen III demonstrated moderate but consistent upregulation across all groups, reflecting balanced ECM deposition.

Tenascin-C and Biglycan are two ECM regulatory proteins essential for remodeling and fibrillogenesis, respectively. Both markers were upregulated by consecutive PBM at day 1 and peaked under NIR PBM at 10 J/cm^2^ on day 4, consistent with their roles in ECM remodeling. Similarly, NIR PBM has been shown to upregulate TNC expression in tenocytes, but BGN has not been evaluated in this context [54]. However, BGN is known to play a role in regulating collagen fibril assembly and maintaining tendon structure; therefore, it is logical to assume that PBM would enhance BGN expression simultaneously when it enhances collagen production [55]. Sustained expression of BGN under green and consecutive PBM by day 10 further highlights their contribution to long-term or graconsecutive ECM remodeling [56].

Together, these findings support a temporal sequence of PBM activity. Near-infrared stimulation promoted early Scx induction and transcriptional priming, consecutive PBM assisted initial ECM synthesis, and green PBM supported late-stage ECM synthesis and remodeling. By the late stage, consecutive and green PBM synergize to enhance Tnmd, Col I, and BGN, consolidating a more mature fibroblastic phenotype. Importantly, the co-expression of SCX and TNMD at day 10, combined with ECM marker regulation, provides strong molecular evidence for tenogenic differentiation of iADMSCs in 3D hydrogel culture [50].

## 4. Materials and Methods

### 4.1. Hydrogel Preparation and Cell Culture

TrueGel3D hydrogel (Sigma-Aldrich, St. Louis, MO, USA, TRUE7) was prepared according to the manufacturer’s instructions and adapted from a 30 µL to a 10 µL hydrogel disk (Table 1). Immortalized ADMSCs (ATCC^®^ SCRC-4000^TM^, Manassas, VA, USA, ASC52Telo hTERT) were encapsulated at a density of 6000 cells/10 µL, and the hydrogels were polymerized in 96-well strip plates (Sigma-Aldrich, BR782301) [57]. Each experimental group and control consisted of four biological repeats (n = 4), as summarized in Table 2. Following encapsulation, cells were cultured for 24 h in complete medium (Dulbecco’s Modified Eagle Medium (DMEM) Gibco; Waltham, MA, USA, 41965-039), supplemented with 10% fetal bovine serum (Gibco, 10493-106) and antibiotics (0.5% Penicillin-Streptomycin [Sigma-Aldrich, P4333], 0.5% Amphotericin B [Sigma-Aldrich, A2942] and 0.5% Gentamycin [Sigma-Aldrich, G1272]).

### 4.2. Tenogenic Differentiation

Immortalized ADMSCs were differentiated using a validated stepwise tenogenic differentiation protocol, consisting of an induction phase followed by a maintenance phase [58]. During induction, cells were cultured in induction medium (complete media supplemented with 10 ng/mL TGF-β_1_ [ThermoFisher; Waltham, MA, USA, 100-21-100UG] and 9 µg/mL L-ascorbic acid [Sigma-Aldrich; A4403]) for 3 days. This was followed by a 7-day maintenance phase, using maintenance medium (induction medium supplemented with 100 ng/mL CTGF [ThermoFisher; 120-19-100UG]). Cultures were maintained at 37 °C, 5% CO_2_ injection, and 85% humidity (Heracell^TM^ 150i CO_2_ incubator, Thermo Scientific, 51026280). The medium was refreshed every other day throughout the experimental period. Assays were conducted on days 1, 4, and 10 post-irradiation, corresponding to the initiation of differentiation (day 1), completion of the induction phase (day 4), and completion of the maintenance phase (day 10).

### 4.3. Photobiomodulation (PBM)

Power output was measured using a FieldMate Laser Power Meter (Coherent, Saxonburg, PA, USA, 1098297), and exposure times required to deliver fluences of 5 and 10 J/cm^2^ were calculated, using Equation (1). The laser parameters are outlined in Table 3. Twenty-four hours after seeding, the complete medium was replaced with 100 µL of induction medium, and cells were allowed to acclimate for 2 h prior to irradiation. Experimental groups were irradiated with either a green diode laser (525 nm, National Laser Centre of South Africa, EN 60825-1:2007 [59]), near infrared diode laser (825 nm, National Laser Centre of South Africa, SN 101080908ADR-1800), or their consecutive (525/825 nm), at 5 or 10 J/cm^2^ fluencies. After irradiation, an additional 130 µL of induction medium was added to each well.

(1) $$mW/{cm}^{2}=\frac{mW}{\pi \times r^{2}}\;W/{cm}^{2}=\frac{mW/{cm}^{2}}{1000}\;Time\left(s\right)=\frac{J/{cm}^{2}}{W/{cm}^{2}}$$

Equation (1): Calculation of the PBM exposure time.

**Table 3:** Laser parameters.

Laser Parameters	Green (G)	Near Infra-Red (NIR)
Light Source	Diode Laser	Diode Laser
Wavelength (nm)	525	825
Power Output (mW)	473	190
Power Density (mW/cm^2^)	52.10	20.93
Emission	Continuous Wave	Continuous Wave
Fluence (J/cm^2^)	5/10	5/10
Time of irradiation (s)	96/192	240/480

### 4.4. Cellular Characterization Using Immunofluorescence

Before differentiation and PBM exposure iADMSCs were characterized for expression of stem cell markers (CD44, CD90, and CD166). The culture medium was removed, cells were washed three times with ice-cold (4 °C) 1× PBS and fixed using 4% paraformaldehyde (PFA) (Sigma-Aldrich, P6148-500G) at room temperature for 15 min. Following fixation cells were washed three times. To prevent non-specific binding, cells were incubated with 5% filtered BSA (Bovine Serum Albumin) (Roche, Basel, Switzerland, 10735086001) in PBS for 30 min at room temperature. Primary antibodies against CD44 (1:100, Sigma-Aldrich, MABF425), CD90 (1:100, Sigma-Aldrich, SAB4200497) and CD166 (1:100, Sigma-Aldrich, ZRB2153) were applied and incubated overnight at 4 °C. After washing, cells were incubated for 2 h at room temperature with the appropriate secondary conjugated antibodies: goat anti-rabbit (1:100, Santa Cruz Biotechnology, Dallas, TX, USA, sc-2359) and goat anti-mouse (1:1000, ThermoFisher, A28175), protected from light. Thereafter, cells were washed, and cell nuclei were counterstained with DAPI (1 µg/µL in PBS) at room temperature for 5 min and washed again. Negative controls were produced by following the same procedure, in the absence of the primary Ab step. Fluorescent images were captured in three respective fields, using the Leica Mica Microhub system (MC-0005026, Leica, Wetzlar, Germany) using the ×10 objective.

### 4.5. Inverted Light Microscopy

Morphology was observed and captured using inverted light microscopy (Olympus CKX41; Tokyo, Japan, SN9B02019) using ×10 magnification.

### 4.6. Recovery Solution

Hydrogel encapsulated cells were recovered using enzymatic digestion (Sigma-Aldrich, TRUEENZ) to dissolve the dextran component of the hydrogel matrix. The reagent was prepared by diluting the stock solution in 1× PBS (1:20). The hydrogels were dissolved using 50 µL of the recovery solution added to the culture media (LDH assay). However, for the ATP and PCR assays, the culture media were removed and discarded before adding the recovery solution. Cell recovery lasted for 60 min at 37 °C, as directed by the manufacturer’s instructions. The subsequent assays were only initiated after confirming complete hydrogel dissolution, which was verified using inverted light microscopy (Olympus CKX41; SN9B02019).

### 4.7. Lactate Dehydrogenase (LDH)

Membrane permeability was assessed by quantifying LDH release into the culture medium using the CytoTox96^®^ Non-Radioactive Cytotoxicity Assay (Promega, Madison, WI, USA, G1780). Briefly, the hydrogels were first dissolved, as specified in the recovery procedure. Thereafter, 50 µL of culture medium was combined with 50 µL of CytoTox96^®^ reagent in a flat-bottomed 96-well clear plates (Corning^®^, Sigma-Aldrich, St. Louis, MO, USA, CLS3370) and incubated for 30 min, protected from light. The reaction was stopped by adding 50 µL of stop solution to each well. The colorimetric compound was quantified by measuring absorbance at 490 nm using the VICTOR Nivo Plate Reader^TM^ (Perkin Elmer, Waltham, MA, USA, HH3522019094). Positive controls were prepared by lysing age-matched controls with 20 µL lysis solution and 25 µL of recovery solution. Background absorbance was subtracted from experimental and control group values.

### 4.8. Adenosine Triphosphate (ATP)

Proliferation was quantified by measuring intracellular ATP levels using the CellTiter-Glo^®^ 3D Cell Viability Assay (Promega, G9681). After removing the culture medium, the hydrogels were dissolved using the recovery solution (50 µL). Followed by the addition of an equal volume of CellTiter-Glo^®^ 3D reagent (50 µL) to each well. Plates were covered with foil and incubated on an orbital shaker (100–150 rpm) for 30 min at room temperature. The resulting lysates were transferred to opaque-walled 96-well plates (Corning^®^, Sigma-Aldrich, CLS3912), and luminescence was measured using the VICTOR Nivo Plate Reader^TM^ (Perkin Elmer, HH3522019094). Background luminescence was subtracted from experimental and control group values. An assay control was prepared using ATP powder (Sigma-Aldrich, A26209-1G) to validate the performance of the ATP kit.

### 4.9. Reverse Transcription Quantitative Polymerase Chain Reaction (RT-qPCR)

Quantitative PCR was used to evaluate the effectiveness of various PBM wavelengths and fluencies in enhancing tenogenic differentiation. Total RNA was extracted using the Quick-RNA^TM^ MiniPrep Plus Kit (Zymo Research, Irvine, CA, USA, ZR R1058). RNA concentration and purity were assessed with a NanoDrop spectrophotometer (Jenway Genova Nano, Essex, UK, 67912), and samples with an A_260_/A_280_ ratio between 1.8 and 2.1 were used for downstream applications. Complementary DNA (cDNA) was synthesized from one microgram of total RNA using the LunaScript^®^ RT SuperMix Kit (New England Biolabs, Ipswich, MA, USA, NEB M3010X), on a Stratagene thermocycler (Agilent Technologies, Santa Clara, CA, USA, Mx3005P). RT-qPCR was performed to determine expression levels for common tenogenic markers (Table 4) using SYBR green Universal qPCR Master Mix (New England Biolabs, M3003E), each quadruplicate sample was run in duplicate (n = 8), on an AriaMx thermocycler (Agilent Technologies, G8830-64001). The thermal cycling program consisted of an initial hot start at 95 °C for 60 s (s), followed by 40 cycles of denaturation at 95 °C for 15 s and primer annealing at 55 °C for 30 s, and melt curve analysis (1 cycle of 95 °C [30 s], to 65 °C [30 s] to 95 °C [30 s]). Gene expression was normalized to GAPDH, confirmed to be stable across experimental conditions, and quantified using the comparative Ct method. Relative expression values were calculated as fold changes, and results were displayed in a heat map to illustrate the degree of change.

### 4.10. Statistical Analysis

All quantitative assays were performed in quadruplicates (n = 4), and all qualitative morphological analyses were performed in duplicates (n = 2). Quantitative data were captured in a Microsoft Excel spreadsheet and analyzed using SigmaPlot version 12 for statistical analysis. The results are expressed as mean and standard error. Experimental groups were compared to the control using a one-way ANOVA to determine significance, defined as *p* < 0.05 (*), *p* < 0.01 (**), and *p* < 0.001 (***). The findings were presented in graphs as mean values with error bars representing the standard error and significance indicated by asterisks.

## 5. Conclusions

In this study, we demonstrated that PBM supported the tenogenic differentiation of iADMSCs encapsulated in TrueGel3D hydrogels through a wavelength-, fluence-, and time-dependent manner, which may inform future PBM optimization studies. MSC characterization confirmed the stemness of the starting population, while morphological analysis revealed a transition to spindle-shaped fibroblastic morphology within the hydrogel. LDH assays showed that PBM and hydrogel encapsulation did not induce cytotoxicity. ATP analysis revealed slower proliferation compared to 2D cultures, consistent with metabolic redirection toward differentiation. Gene expression analysis supported tenogenic lineage identification.

A single dose approach using consecutive (525/825 nm) wavelengths at 10 J/cm^2^ proved effective in enhancing tenogenic marker expression. However, as a future directive, the temporal data observed suggests that a sequential PBM strategy may be more effective. In theory, such an approach aligns with the stepwise biochemical differentiation protocol employed in this study, focusing on lineage commitment (day 1–3 using TGF-β_1_) and thereafter matrix maturation (day 4–10 using CTGF). Therefore, to maximize PBM efficacy, we propose a sequential irradiation protocol in which NIR (10 J/cm^2^) PBM is applied first to promote transcriptional priming and ECM remodeling (day 1–4), followed by green (5 J/cm^2^) or consecutive (10 J/cm^2^) PBM from day 4 to enhance tenocyte maturation and fibrillogenesis (day 4–10). Integration of this staged PBM protocol with scaffold-based strategies may further enhance tendon regeneration.

This study had certain limitations. Hydrogel constraints, specifically the random distribution of RGD peptides, limited cellular alignment and prevented the anisotropic organization characteristic of native tendon tissue. Furthermore, the short-term culture period of 10 days captured early-to-intermediate differentiation events and may not fully represent long-term matrix maturation. Additionally, the use of immortalized cell lines limits the findings to in vitro laboratory work. Future studies should combine aligned scaffolding and longer culture periods for in vitro studies. Additionally, future studies should validate transcriptional responses at protein and functional levels, particularly using primary cells in in vivo models to assess structural organization and biomechanical outcomes.

## Figures and Tables

**Table 1:** Hydrogel components and quantities.

Component	Volume
Water	2 µL
Buffer	0.8 µL
Dextran	1.7 µL
RGD peptide	1.3 µL
Cell suspension	1.7 µL
Cross linker	2.5 µL
Total	10 µL

**Table 2:** Summary of experimental groups.

Experimental Group	PBM Treatment	Growth Factors
Control	None	Tenogenic differentiation
525 nm	525 nm; 5 J/cm^2^	
	525 nm; 10 J/cm^2^	
825 nm	825 nm; 5 J/cm^2^	
	825 nm; 10 J/cm^2^	
525/825 nm (consecutive)	525/825 nm; 5 J/cm^2^	
	525/825 nm; 10 J/cm^2^	

**Table 4:** List of PCR primers.

Target Gene	Forward Primer	Reverse Primer	Annealing Temperature	Amplicon Size	Accession Number	Reference to Accession Number
*Scleraxis* (*Scx*)	AGAACACCCAGCCCAAAC	CCACCTCCTAACTGCGAATC	55 °C	102	NM_001080514.3	[60]
*Tenomodulin* (*Tnmd*)	GATCCTGTGACCAGAACTGAAA	CGAAGTAGATGCCAGTGTATCC	55 °C	100	NM_022144.3	[61]
*Collagen I alpha 1 chain* (*Col 1A1*)	CCTGTCTGCTTCCTGTAAACTC	GTTCAGTTTGGGTTGCTTGTC	55 °C	101	NM_000088.3	[62]
*Collagen 3 alpha 1 chain* (*Col 3A1*)	GAGTCCATGGATGGTGGTTT	CTGGAGAGAAGTCGAAGGAATG	55 °C	98	NM_000090.3	
*Biglycan* (*BGN*)	CTCGTCCTGGTGAACAACAA	CAGGTGGTTCTTGGAGATGTAG	55 °C	96	NM_001711.6	[63]
*Tenascin-C* (*TNC*)	GATGCCAAGACTCGCTACAA	GTCAAAGGTGGAGAAGGATCTG	55 °C	99	NM_002160.4	[64]
*GAPDH*	CAAGAGCACAAGAGGAAGAGAG	CTACATGGCAACTGTGAGGAG	55 °C	102	NM_002046.3	

## Data Availability

The original contributions presented in this study are included in the article. Further inquiries can be directed to the corresponding author.

## Abbreviations

The following abbreviations are used in this manuscript:

µg	Microgram
µg/mL–µg	Microgram per milliliter
ADMSCs	Adipose-derived mesenchymal stem cells
ATCC	American Type Culture Collection
ATP	Adenosine triphosphate
BGN	Biglycan
CD	Cluster of differentiation
Col I	Collagen I
Col III	Collagen III
CTGF	Connective tissue growth factor
DAPI	4′, 6-Diamidino-2-phenylindole
DMEM	Dulbecco’s Modified Eagle Medium
ECM	Extracellular matrix
FBS	Fetal bovine serum
iADMSCs	Immortalized adipose-derived mesenchymal stem cells
J/cm^2^	Joules per square centimeter
LDH	Lactate dehydrogenase
MSCs	Mesenchymal stem cells
ng/mL	Nanograms per milliliter
NIR	Near-infrared
nm	Nanometer
PBM	Photobiomodulation
PBS	Phosphate-buffered saline
PCR	Polymerase chain reaction
PFA	Paraformaldehyde
Scx	Scleraxis
TGF-β_1_	Transforming growth factor β_1_
TNC	Tenascin-C
Tnmd	Tenomodulin
